# Recent Development of Hydrogen Sulfide Releasing/Stimulating Reagents and Their Potential Applications in Cancer and Glycometabolic Disorders

**DOI:** 10.3389/fphar.2017.00664

**Published:** 2017-09-26

**Authors:** Chun-tao Yang, Li Chen, Shi Xu, Jacob J. Day, Xiang Li, Ming Xian

**Affiliations:** ^1^Affiliated Cancer Hospital and Institute, Key Laboratory of Protein Modification and Degradation in School of Basic Medical Sciences, Guangzhou Medical University, Guangzhou, China; ^2^Department of Chemistry, Washington State University, Pullman, WA, United States

**Keywords:** hydrogen sulfide, donor, cancer, glucose metabolism

## Abstract

As an important endogenous gaseous signaling molecule, hydrogen sulfide (H_2_S) exerts various effects in the body. A variety of pathological changes, such as cancer, glycometabolic disorders, and diabetes, are associated with altered endogenous levels of H_2_S, especially decreased. Therefore, the supplement of H_2_S is of great significance for the treatment of diseases containing the above pathological changes. At present, many efforts have been made to increase the *in vivo* levels of H_2_S by administration of gaseous H_2_S, simple inorganic sulfide salts, sophisticated synthetic slow-releasing controllable H_2_S donors or materials, and using H_2_S stimulating agents. In this article, we reviewed the recent development of H_2_S releasing/stimulating reagents and their potential applications in two common pathological processes including cancer and glycometabolic disorders.

## Introduction

For a long time, hydrogen sulfide (H_2_S) was considered to be a colorless, flammable, water-soluble, and highly toxic environmental hazard with the characteristic smell of rotten eggs. However, research conducted over the last decade suggests that this gaseous molecule can be endogenously generated and exerts very important roles in organisms (Szabo, 2007, 2012; Wang, 2012; Lv et al., 2017). In mammals, endogenous H_2_S is mainly produced through the metabolism of L-cysteine and homocysteine by the catalysis of two pyridoxal-5′-phosphate (PLP)-dependent enzymes, cystathionine β-synthase (CBS) and cystathionine γ-lyase (CSE) (Chiku et al., 2009; Singh et al., 2009). It can also be generated from the PLP-independent 3-mercaptopyruvate sulfurtransferase (3-MST), or cysteine aminotransferase (CAT) in the presence of α-ketoglutarate. The optimum pH value of 3-MST and CAT for the catalytic H_2_S production is about 9.7, while the physiological pH value of human blood ranges from 7.35 to 7.45. Additionally, 3-mercaptopyruvate, a substrate of 3-MST, is quite unstable. Therefore, the biological significance of this enzymatic reaction remains controversial, especially under physiological conditions (Mikami et al., 2011; Yadav et al., 2013).

These H_2_S-producing enzymes are distributed throughout the body in a tissue-specific manner. In murine liver and kidney tissues, CBS and CSE are both expressed, but the protein level of CSE is significantly higher than that of CBS. At high substrate concentrations (up to 20 mM cysteine or homocysteine) the capacity for liver H_2_S production is approximately equal for CBS and CSE, whereas in the kidney CBS constitutes the major source of H_2_S (Kabil et al., 2011). A number of studies have revealed that H_2_S can exert a variety of biological effects. For example, H_2_S is able to reduce vascular tone and stimulate angiogenesis (Lefer, 2007; Polhemus and Lefer, 2014). The genetic deletion of CSE in mice decreased H_2_S generation and led to pronounced hypertension and impaired endothelium-dependent vasorelaxation (Yang et al., 2008). In inflammatory response, H_2_S seems to be a double-edged sword. It can reduce inflammation and promote tissue repair by targeting many elements of the inflammatory cascade (Wallace et al., 2015) whereas in endotoxic shock, H_2_S can trigger or facilitate inflammation response in the lungs (Li et al., 2005). All of these findings suggest that the regulation of H_2_S content in the body may have great therapeutic value.

To understand the functions of H_2_S and develop H_2_S-related therapy, reagents that can be used to produce H_2_S *in vitro* and *in vivo* are often needed. Those compounds are known as H_2_S donors or stimulating reagents. The development of such chemicals has received much attention from medicinal chemists and chemical biologists. A number of different H_2_S donors have been reported and the topic of H_2_S donors have been reviewed several times (Li et al., 2008; Zhao et al., 2014; Steiger et al., 2016; Zhao and Pluth, 2016; Zheng et al., 2016). Herein, we provide an overview on current understanding of commonly used H_2_S donors and stimulating reagents. We focus our discussion on recent development of H_2_S donors, donor materials, and stimulating reagents. It is worthwhile to note that cancer and glycometabolic disorders have become an increasing public health concern throughout the world. Recent studies have revealed some unique functions of H_2_S in these diseases. Therefore, in this article we also reviewed the studies and results of applying H_2_S in these pathophysiological processes.

## Donors of Hydrogen Sulfide

### Gaseous H_2_S

H_2_S gas can be inhaled by testing animals. Therefore, experiment animals can be put into an H_2_S-riched environment to observe H_2_S’s physiological effects or toxicity. For example, it was found that when mice were exposed to 80 ppm of H_2_S for 6 h, their oxygen consumption dropped by ∼50%, and the metabolic rate and core body temperature were also significantly decreased into a suspended animation state (Blackstone et al., 2005). This effect is associated with the inhibition of cytochrome C oxidase of the electron transport chain during oxidative phosphorylation (Beauchamp et al., 1984). Notably, lowering metabolic demand could be useful for the reduction of physiological damage caused by trauma and improve outcomes after surgery (Blackstone et al., 2005). However, a later study of various larger species, such as sheep, swine, and human, indicated that H_2_S only exerted thermoregulatory effects (Wagner et al., 2011). H_2_S has good solubility in water (110 mM/atm at room temperature; 210 mM/atm at 0°C). Therefore, solutions of H_2_S gas are often used in studies. For example, in type 2 diabetes H_2_S gas solutions were used and it was found that they could promote glucose uptake through amelioration of insulin resistance and reduce renal injury (Xue et al., 2013). It should be noted that solutions with precise H_2_S concentrations are difficult to obtain, as H_2_S gas can easily escape from the solutions leading to a decreased concentration. In addition, H_2_S is a highly toxic gas, especially at high concentrations. These problems limit the use of H_2_S gas as a suitable reagent for many researchers.

### Inorganic Sulfide Salts

Under physiological pH, H_2_S is in fast equilibrium with HS^-^ in aqueous solutions. The proportions of HS^-^ and H_2_S are 81 and 19%, respectively. Therefore, inorganic sulfide salts, such as sodium hydrosulfide (NaHS) and sodium sulfide (Na_2_S), are often used as H_2_S equivalents in many studies. These salts are easy to obtain and widely used in the preparation of H_2_S solutions. However, these salts are considered to be fast H_2_S donors, as they produce H_2_S immediately when dissolved in aqueous solutions. Moreover, H_2_S molecule can rapidly escape from the buffers under a variety of experimental conditions, such as in the studies of tissue culture plates, muscle myograph baths, and Langendorff perfused heart apparatus (DeLeon et al., 2012). This loss of H_2_S is mainly due to the rapid volatilization of H_2_S. This problem may explain the discrepancy between low H_2_S concentrations in blood and tissues versus high concentrations of exogenous H_2_S (when sulfide salts are used) required to produce physiological responses (DeLeon et al., 2012). When exposed to high concentrations of H_2_S for a short period of time, tissues and cells may be damaged or show different responses, therefore, it is hard to investigate the effects of physiological concentrations of H_2_S (at low μM or nM levels). Another problem is that H_2_S tends to oxidize, particularly in the presence of metal contaminants in solutions. It is therefore recommended to prepare solutions in anaerobic water/buffers. The addition of heavy metal chelators, like DTPA (diethylenetriaminepentaacetic acid), can chelate metal ions and stabilize H_2_S in solutions. The use of DTPA is highly recommended when working with H_2_S solutions.

Nevertheless, NaHS has been used as a standard H_2_S donor in many studies. For example, it was demonstrated that NaHS could alleviate amyloid beta-peptide (Aβ) _25-35_-induced neural lesion in an Alzheimer’s disease cellular model (Tang et al., 2008). In doxorubicin-induced cardiotoxicity, NaHS was found to reduce cardiomyocyte injury through inhibition of endoplasmic reticulum (ER) stress (Wang et al., 2012). In hypoxic skin damage, NaHS could exert anti-inflammatory effects through inhibition of reactive oxygen species (ROS)-activated nuclear factor kappa B (NF-κB)/cyclooxygenase (COX)-2 (Yang et al., 2011). Meanwhile, some researchers prefer using Na_2_S as the H_2_S donor. For example, Lefer and colleagues suggested that Na_2_S could attenuate mouse myocardial ischemia-reperfusion injury by preservation of mitochondrial function (Elrod et al., 2007). In another study on inflammatory modulation, the administration of Na_2_S (28 μmol/kg) was proved to suppress carrageenan-induced rat paw edema by producing H_2_S (Zanardo et al., 2006). Notably, the intravenous administration of Na_2_S (0.005–0.20 mg/kg, infused over 1 min) to healthy human volunteers increased blood sulfide and thiosulfate concentrations, as well as exhaled H_2_S gas (Toombs et al., 2010). Clinically, the elevated amount of exhaled H_2_S gas by patients was used to predict a non-eosinophilic phenotype of chronic obstructive pulmonary disease (Zhang et al., 2015) and the airway inflammation of asthma (Zhang et al., 2014).

### Garlic-Derived Sulfur-Containing Compounds

Garlic is rich in sulfur-containing compounds and can be considered as an active “H_2_S” pool. Allicin (diallyl thiosulfinate) is the most well-characterized compound in garlic. In aqueous solutions allicin decomposes to a number of reactive sulfur-containing compounds, including diallyl sulfide (DAS), diallyl disulfide (DADS), and diallyl trisulfide (DATS) (Amagase, 2006), which can release H_2_S in different manners. DATS reacts rapidly with GSH to release H_2_S through thiol-disulfide exchange followed by allyl perthiol reduction by GSH. However, DADS only releases a minute amount of H_2_S via a sluggish reaction with GSH through an alpha-carbon nucleophilic substitution pathway (Liang et al., 2015). In human erythrocytes, garlic-derived organic polysulfides could be converted into H_2_S through formation of a key intermediate, hydropolysulfide (RS_n_H) (Benavides et al., 2007). In recent years, DADS and DATS have often been used as H_2_S donors to investigate their biologic actions. For example, it was shown that DADS can inhibit tumor cell proliferation and improve tissue repair (Ciocci et al., 2016). Since this class of H_2_S donors have been used clinically, the donation of H_2_S from them may have vital clinical significance.

### Synthetic Slow-Releasing H_2_S Donors

**FIGURE 1:**
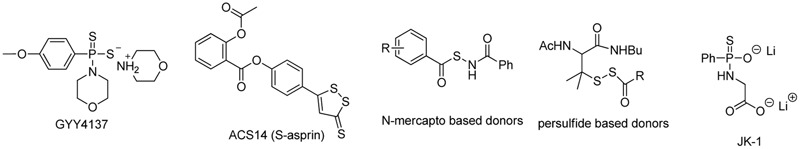
The structures of representative synthetic H_2_S donors.

Given the problems encountered with using H_2_S gas or sulfide salts in studies, researchers have explored a number of synthetic small molecules as H_2_S-releasing agents, e.g., H_2_S donors. These compounds are usually stable in physiological buffers. When applied to biological systems, they can release H_2_S under different mechanisms, such as hydrolysis, photolysis, or via the interaction with biomolecules like biothiols or enzymes. Some representative synthetic H_2_S donors are shown in **Figure 1**. GYY4137, a Lawesson’s reagent derivative, is perhaps the most well-known H_2_S donor. It has been used by many researchers as the standard slow H_2_S production source. The release of H_2_S from GYY4137 is very slow in aqueous solutions as well as after intravenous or intraperitoneal administration in animals (Li et al., 2008; Chen et al., 2016). GYY4137’s H_2_S release mechanism is suggested to be due to direct hydrolysis or interacting with un-identified biomolecules but the details still need to be elucidated. A sulfur-containing heterocycle dithiolethione is another often-used H_2_S-releasing motif. It has been used particularly in the construction of hybrid drugs with non-steroidal anti-inflammatory drugs (NSAIDs). H_2_S-releasing NSAIDs are designed with the primary goal of using H_2_S to overcome gastric side-effects of traditional NSAIDs through improvement of blood flow, attenuation of oxidation and inflammation (Liu et al., 2012). A series of H_2_S-releasing NSAIDs have been developed, such as HS-aspirin (ACS14) (**Figure 1**), HS-sulindac, HS-ibuprofen, HS-naproxen, S-sulindac, S-ibuprofen, and S-naproxen, and their inhibitory effects on cancer cells have also been described in detail (Chattopadhyay et al., 2012; Kodela et al., 2015b; Vannini et al., 2015; De Cicco et al., 2016). Besides the gastric protection and the effects against cancer, these hybrids have shown more potentials for relieving inflammatory pain than the traditional NSAIDs (Fonseca et al., 2015). However, to date, it is still unclear how H_2_S is generated from dithiolethiones nor is the by-product known. Therefore, this lack of information may be a problem in developing these compounds for clinic uses.

H_2_S release from the aforementioned donors is considered non-controllable, as the release is either due to the donor’s spontaneous hydrolysis or the mechanism is still unknown. Recently, some controllable H_2_S donors have been developed. The representative examples are persulfide-based donors (Zhao et al., 2013), *N*-mercapto-based donors (Zhao et al., 2015), and JK type donors (Kang et al., 2016; Yang et al., 2017a), which are also shown in **Figure 1**. These are considered “controllable” donors because their H_2_S release mechanisms are clearly exploited and the structural modifications could lead to H_2_S release profile changes (faster or slower). As such, these compounds can potentially be used for precisely delivering H_2_S in biological systems. Nevertheless these synthetic donors have been reviewed multiple times. Interested readers can refer to those review articles (Zhao et al., 2014; Zheng et al., 2017). Herein we would like to focus on some recent developments in this field.

### Ammonium Tetrathiomolybdate

**FIGURE 2:**
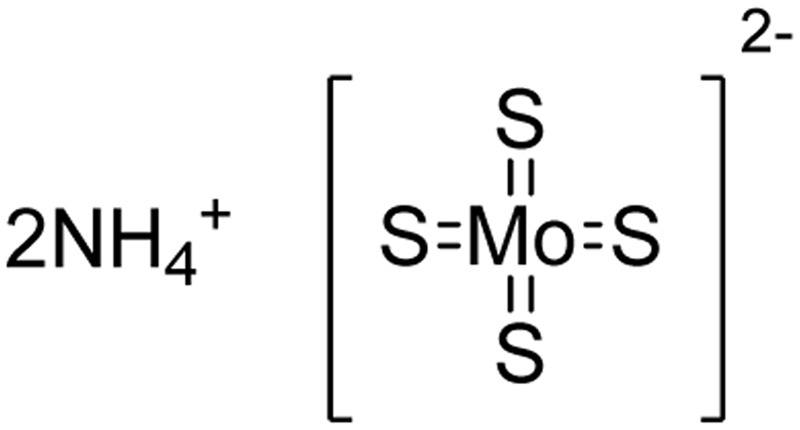
The chemical structure of TTM.

Thiomolybdate salts have been used as thiol transfer reagents in organic synthesis (Prabhu et al., 2002). The four sulfur atoms in the structures also make them excellent copper chelators. Ammonium tetrathiomolybdate (TTM) (**Figure 2**) has been used clinically as a treatment for copper toxicity, especially for Wilson’s disease (Brewer, 2003), a genetically recessive disease of copper accumulation. TTM’s pharmacology and toxicology have been thoroughly studied (Brewer, 2009). Also, it is known that TTM can produce H_2_S under strong acidic conditions such as 5% H_2_SO_4_ (Wang et al., 1997). As such, it is possible to repurpose TTM as a unique inorganic complex-based H_2_S donor, analogous to the discovery of sodium nitroprusside as a widely used nitric oxide (NO) donor (Hottinger et al., 2014). To this end, we studied TTM’s H_2_S-generation and relevant activities under physiological conditions (Xu et al., 2016). Using a modified methylene blue method, commercially purchased TTM (99.97% purity from Sigma-Aldrich) was used to test H_2_S release in phosphate-buffered saline under pH 5, 6, 7.4, and 8. The results showed that TTM released H_2_S upon solvation, and H_2_S concentrations in all pH levels were maintained at a steady level for up to 15 h. These results suggest that TTM is a slow H_2_S releaser, similar to GYY4137. It was also found that acidic pH could accelerate TTM’s H_2_S release. Further studies in cellular models showed TTM could protect cells under oxidative stress (treated by H_2_O_2_). These preliminary results indicate TTM is a promising H_2_S donor (Xu et al., 2016). A recent study has shown that TTM could attenuate myocardial and cerebral injury induced by ischemia/reperfusion (Dyson et al., 2017). More studies on it are expected to come in the next years.

### Reactive Oxygen Species Activated H_2_S Donors

**FIGURE 3:**
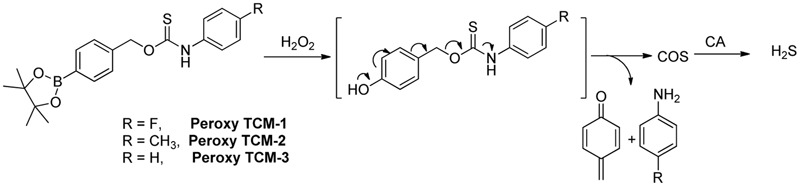
H_2_O_2_-mediated COS/H_2_S release from Peroxy TCM donors.

In 2016, Pluth and colleague designed several interesting ROS activated H_2_S donors (Zhao and Pluth, 2016). These caged thiocarbamate compounds bear a pinacol boron functional group, which can be oxidized and cleaved to hydroxyl (-OH) by ROS, like hydrogen peroxide (H_2_O_2_). A tandem reaction will then occur and release carbonyl sulfide (COS), as well as quinone and amine byproducts (**Figure 3**). It is worth noting that COS can easily undergo hydrolysis to produce H_2_S if carbonic anhydrase (CA) is presented (Steiger et al., 2016). This work represents the first ROS triggered H_2_S donors. It was found that the reaction rates could be tuned by electronic modulation of thiocarbamates. Interestingly, further studies showed CA is not required for the donors’ H_2_S release as H_2_O_2_ can also trigger the rapid transformation of COS to H_2_S. Cellular imaging experiments demonstrated the donors, such as Peroxy TCM, can release H_2_S with activation of both exogenous H_2_O_2_ and endogenous H_2_O_2_ in cells. The donors can increase cell viability under H_2_O_2_-induced oxidative stress, while the control compounds without aryl boron moiety, which produces H_2_O instead of H_2_S, show little or no protective effects against H_2_O_2_ induced damage. These results indicate that these donors are effective under ROS stimulation.

### Enzyme Activated H_2_S Donors

**FIGURE 4:**
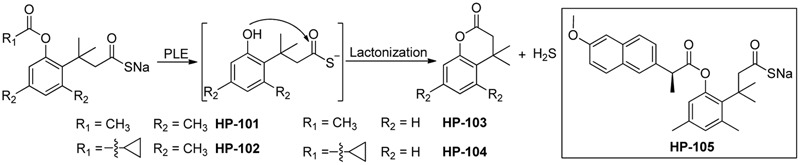
H_2_S release from esterase-activated donors.

Also in 2016, Wang and colleagues reported a series of esterase activated H_2_S donors (**Figure 4**) (Zheng et al., 2016). These donors contain a thioacid moiety and an ester-masked nucleophilic phenol in a “trimethyl lock” template. After the ester group is removed by an esterase, a spontaneous intramolecular lactonization occurs, resulting in the release of H_2_S. Experimental data showed the donors require porcine liver esterase or cell culture media containing FBS to release H_2_S, confirming the donors’ selectivity and chemical stability. Additionally, their H_2_S-releasing rates can be tuned by adjusting ester substituents and the trimethyl lock structure. In RAW 264.7 cells, these donors inhibited lipopolysaccharide-induced tumor necrosis factor (TNF)-α secretion, suggesting the H_2_S-related anti-inflammatory effects, while control compounds without the H_2_S-releasing moiety did not show such effects. These esterase activated donors can be conjugated to NSAIDs to form H_2_S–NSAID hybrids. Such hybrids, like HP-105, could utilize H_2_S’s anti-inflammatory and anti-oxidative effects to ameliorate NSAIDs-induced gastric damage. However, detailed biological evaluations of these new H_2_S–NSAID hybrids have yet to be seen.

**FIGURE 5:**
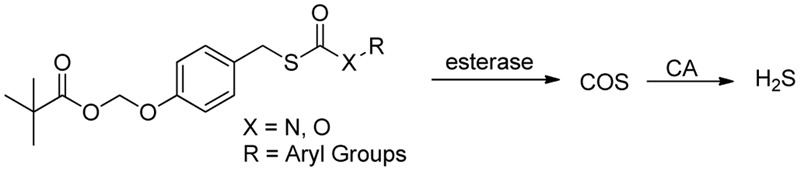
Esterase-activated COS/H_2_S release.

Besides Wang’s work (Zheng et al., 2016), Chakrapani and colleagues also designed esterase-activated H_2_S donors (**Figure 5**), utilizing carbamothioates and carbonothioates to generate COS as the H_2_S precursor (Chauhan et al., 2017). COS is then hydrolyzed by CA to produce H_2_S. These donors’ ability of generating H_2_S when esterase and CA are both presented has been proven by various studies (Steiger et al., 2017a,b). Kinetics studies showed the decomposition of the compounds may go through a short-lived intermediate.

**FIGURE 6:**
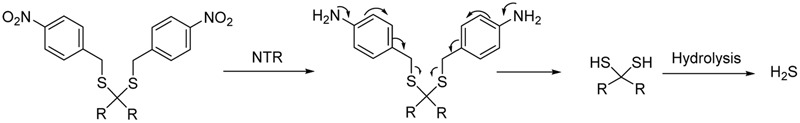
Nitroreductase-activated H_2_S donors.

Nitroreductase (NTR)-activated H_2_S donors were recently reported by Chakrapani and colleagues (Shukla et al., 2017). The donors contain 4-nitroaryl masking groups, which are good substrates for NTR. Upon nitro reduction, a *gem*-dithiol will be produced, and further converted to H_2_S (**Figure 6**). Fluorescent and monobromobimane assays proved the donors’ H_2_S generation in cell-free conditions in the presence of bacterial NTR. The donors also generate H_2_S in bacterial cells that express NTR, but fail to generate H_2_S in bacterial cells or mammalian cells deficient in NTR. These donors were used as chemical tools to study H_2_S’s role in antimicrobial resistance.

These ROS- and enzyme-activated donors utilize interesting chemistry, and make their H_2_S release more controllable. They can be useful chemical tools. However, there are very few studies about their biological applications or therapeutic potentials. We look forward to these studies in the near future.

### H_2_S-Releasing Materials

Compared to small molecule drugs, material-based drugs have some unique advantages, such as improved water solubility and stability, slower clearance rates, and reduced toxicity. Material-based drug delivery has been extensively studied. However, H_2_S-releasing biomaterials are relatively new in this field. In the past several years, a few such materials have been developed, which are summarized below.

**FIGURE 7:**
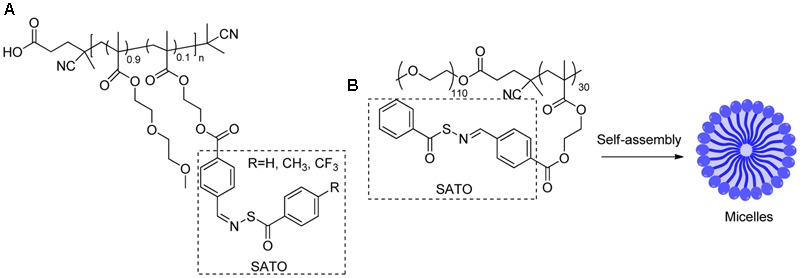
**(A)** SATO based polymers. **(B)** SATO based micelles.

In 2014, Matson and colleague prepared *S*-aroylthiooxime (SATO)-based polymers A (**Figure 7**) by functionalizing aldehyde-containing polymers with *S*-aroylthiohydroxylamines (Foster and Matson, 2014). In the presence of biological thiols, such as cysteine or GSH, the SATO moiety on the polymer can degrade to release H_2_S. The kinetics can be controlled by tuning electronics on SATO substituents. Recently, the same strategy was also employed to prepare SATO-based micelles by the same group (Foster et al., 2017). In this case, SATO was formed on polymer amphiphiles to give B, which undergoes self-assembly in THF-water solutions and forms micelles. Similarly, these micelles release H_2_S upon activation by biothiols. The micelles showed slower but more sustained H_2_S release than A and small molecule SATOs. This could be attributed to the more difficult diffusion of biothiols to the hydrophobic cores of micelles. In the presence of cysteine, the H_2_S-micelles showed cytotoxicity toward cancer cells, such as HCT 116 cells, but much reduced toxicity for “normal” cells such as NIH/3T3 cells, suggesting that H_2_S-micelles can selectively target cancer cells.

**FIGURE 8:**
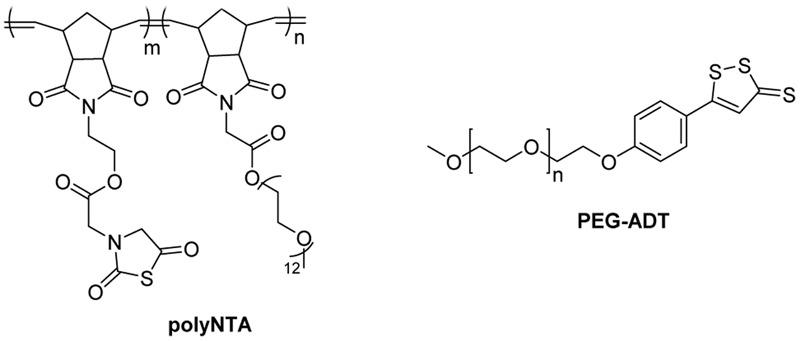
The structures of polyNTA and PEG-ADT.

In 2016, Matson’s group developed polyNTA (**Figure 8**), a random polymer that can release H_2_S via the COS intermediate (Powell et al., 2016). This polymer contains *N*-thiocarboxyanhydrides (NTAs), which undergoes a ring-opening reaction with biological nucleophiles, such as amines to release COS. Then COS can be hydrolyzed to produce H_2_S in the presence of CA. PolyNTA was shown to generate H_2_S in buffers when glycine and CA were present. However, it failed to promote endothelial cell proliferation like small molecule H_2_S donors.

Another example of an H_2_S-relasing polymer is PEG-ADT (**Figure 8**) developed by van der Vlies and colleague (Hasegawa and van der Vlies, 2014). In this work, H_2_S donor 5-(4-hydroxyphenyl)-3H-1,2-dithiole-3-thione (ADT-OH) was conjugated to polyethylene glycol (PEG). PEG-ADT showed lower H_2_S generating capacity than its parent compound ADT-OH and reduced cytotoxicity. Cell imaging studies suggested PEG-ADT could enter cells via endocytosis and remain in the endolysosome, whereas ADT-OH enters the cytoplasm via membrane diffusion. This difference may lead to reduced toxicity of PEG-ADT.

Apart from chemical conjugations, Wang et al. (Feng et al., 2015; Wu et al., 2016b) reported H_2_S-releasing nanofibers by physically adding H_2_S donor molecules to polycaprolactone (PCL) solutions and fabricated the materials with electrospinning. Two H_2_S-releasing nanofibers, thiol-activated H_2_S-fibers (Feng et al., 2015) and pH-activated PCL-JK1 (Wu et al., 2016b) were prepared. These fibers showed prolonged H_2_S release compared to their parent small molecules. Notably, they also showed significant cytoprotection and improved wound healing capacity.

### SG-1002

**FIGURE 9:**
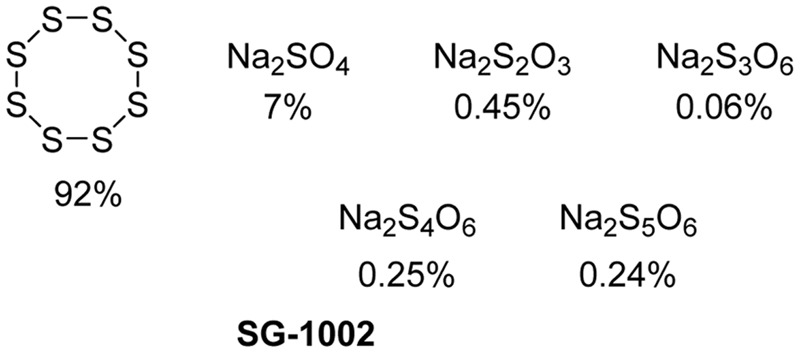
SG-1002 Components.

SG-1002 (**Figure 9**) is an inorganic mixture, containing S_8_, Na_2_SO_4_, Na_2_S_2_O_3_, Na_2_S_3_O_6_, Na_2_S_4_O_6_, and Na_2_S_5_O_6_. It was developed by SulfaGENIX as an oral H_2_S donor for the treatment of heart diseases, like myocardial I/R injury and heart failure. In a pre-clinic study, SG-1002 effectively restored H_2_S and sulfane sulfur levels in mice with heart failure triggered by transverse aortic constriction (TAC) (Kondo et al., 2013). SG-1002 treated mice suffered less cardiac enlargement and left ventricular (LV) dilation, and showed reduced fibrosis compared with mice fed by a control diet after TAC. Additionally, the treatment with SG-1002 upregulated the vascular endothelial growth factor (VEGF), protein kinase B (Akt), endothelial NO synthase, NO and cGMP pathway, and attenuated mitochondrial respiratory dysfunction and oxidative stress induced by TAC.

In another model study, SG-1002 attenuated cardiac dysfunction induced by a high fat diet (HFD) in mice (Barr et al., 2015). HFD fed mice showed decreased sulfide levels, metabolic perturbation such as increased serum glucose level and glucose intolerance, as well as cardiac dysfunction such as decreased LV function fraction and increased circumferential stress. Morphological histological analysis showed cardiac enlargement and fibrosis in HFD fed mice. However, the treatment with SG-1002 markedly ameliorated some of the metabolic perturbations and attenuated cardiac dysfunction. Further study showed that the treatment could restore adiponectin levels and suppress cardiac ER stress.

In a Phase I clinical trial (Polhemus et al., 2015), the effects of SG-1002 were tested on both healthy subjects and subjects with heart failure with dosages up to 800 mg twice daily for 7 days. Both groups showed increased H_2_S levels with only minor adverse effects observed. In addition, a double blind, placebo controlled Phase I trial (ClinicalTrials.gov Identifier: NCT01989208) is in process.

Despite the promise, no present study has described the H_2_S releasing mechanism of SG-1002 *in vivo*. We look forward to studies that elucidate the mechanism, which should provide new insight in H_2_S-related therapies.

### H_2_S-Stimulating Agents

In addition to donors that directly degrade to produce H_2_S via different chemical mechanisms, some compounds are known to stimulate H_2_S production *in vivo*. For example, L-cysteine is an important natural substrate of H_2_S-producing enzymes. When the amino group of L-cysteine is acetylated, the resulted product *N*-acetyl-L-cysteine (NAC) is one of the essential medicines listed by the World Health Organization (WHO). Although NAC has been used clinically as an expectorant (Sheffner, 1963) or an antioxidant in hepatic failure (Saito et al., 2010), no clinical trial has used it as a H_2_S donor. However, NAC was found to suppress leukocyte infiltration in an air pouch model through generation of endogenous H_2_S (Zanardo et al., 2006).

**FIGURE 10:**
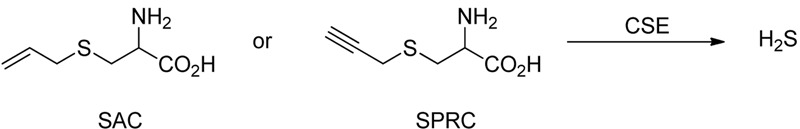
The Structures of SAC and SPRC and their H_2_S release reaction.

*S*-allyl-L-cysteine (SAC) (Kim et al., 2006) and *S*-propargyl-L-cysteine (SPRC) (Gong et al., 2011) (**Figure 10**) are two other cysteine derivatives that can be used as CSE substrates to produce H_2_S. The classical effects of H_2_S including antioxidation and cardioprotection were demonstrated in SAC treated rats suffering from isoproterenol toxicity (Padmanabhan and Prince, 2006). Zhu and colleagues further found that SAC’s cardioprotective effects were associated with induction of CSE expression and enhancement of H_2_S generation (Chuah et al., 2007). A recent study showed that SAC could suppress hepatocyte growth factor-induced migration and invasion in nasopharyngeal cancer cells (Cho et al., 2015). SAC could also exert anti-diabetic effects by means of improving beta-cell function and reducing glycemia (Kim et al., 2017).

SPRC is a structural analog of SAC and has expected similar effects as SAC, such as enhanced CSE expression and plasma H_2_S concentrations, as well as cardioprotection (Wang et al., 2009) and anti-cancer effects (Ma et al., 2011). Recently, it was documented that SPRC could prevent doxorubicin-induced cardiotoxicity (Wu et al., 2016a), which is similar to the effects of NaHS used in previous studies (Wang et al., 2012). In addition, SPRC exhibited anti-inflammation (Wang et al., 2016), anti-apoptosis and anti-oxidation in high glucose treated cardiomyocytes (Yang et al., 2015).

Vitamin D (VD), a key endogenous hormone, has been thought to regulate calcium and phosphate metabolism contributing to bone growth and remodeling for a long time. In recent years, the extraskeletal effects of VD have been documented, such as cancer progression, cardiovascular action, and immune regulation (Christakos et al., 2016). In fact, H_2_S can exert some coincident effects in the body with VD. Notably, Wilinski et al. (2012) found that cholecalciferol, also known as VD_3_, could increase tissue H_2_S concentration in mouse heart, brain and kidney. Meanwhile, another report suggested that VD_3_ had ability to upregulate glucose transporter type 4 (GLUT4) and decreased glycemia in diabetes through induction of CSE expression and H_2_S generation (Manna and Jain, 2012). Moreover, in high glucose and methylglyoxal (MGO)-induced diabetic peripheral neuropathy, VD could exert significant protective roles through restoration of CBS expression and endogenous H_2_S formation (Zhang et al., 2016).

**Table 1** provides a brief summary of H_2_S donors and stimulating reagents covered in this review.

**Table 1:** Summary of H_2_S releasing/stimulating reagents.

Reagents	Potential applications	Advantages	Disadvantages
Gaseous H_2_S	Reduce body temperature and metabolic rate; improve glucose uptake in type 2 diabetes	Clean H_2_S source; no byproducts; easy administration; good absorbability	High volatility; difficult to control concentrations; uncontrolled H_2_S release
**Inorganic sulfide salts**			
NaHS, Na_2_S	Widely used in cellular and animal disease models; intravenous administration to healthy volunteers	Good solubility; easy to handle; clean H_2_S source, no byproducts	High volatility; difficult to control concentrations; Uncontrolled H_2_S release
**Garlic-derived sulfur containing compounds**			
DAS, DADS, DATS	Proliferative inhibition in tumor cells; improvement of tissue repair and myocardial function	Extensive data on a variety of models	Stability of polysulfides is a concern; interfere with GSH
**Synthetic slow-releasing H_2_S donors**			
GYY4137, ACS14	Cytoprotection; anti-cancer and anti-inflammation activities	Good stability; slow H_2_S release; ACS14 overcomes side effects of NSAIDs	Uncontrolled release; unclear release mechanism; produce byproducts
**Novel controllable H_2_S donors**			
*N*-mercapto/persulfide-based donors, JK donors	Myocardial/gastric preservation	Good stability; controlled H_2_S release	Produce byproducts
Tetrathiomolybdate	A common Cu^2+^ chelator; dermal, myocardial and cerebral preservation	Good stability and safety; a clinically used drug	Unclear byproducts could cause side effects
ROS-/enzyme-activated H_2_S donors	Cytoprotection and anti-inflammation	Good stability; controlled H_2_S release	Produce byproducts; lacking *in vivo* data
**H_2_S-releasing materials**			
SATO-based polymers/micelles, polyNTA, PEG-ADT, PCL-JK1	Anti-cancer activity; improvement of wound healing; cardioprotection	Good solubility, stability, membrane permeability; slow clearance rates	More *in vivo* data are needed
**H_2_S-stimulating agents**			
L-cysteine derivatives, Vitamin D	Anti-cancer, anti-diabetes, anti-oxidation, anti-inflammation, and cardioprotection	Transformed into endogenous substances	

From the above sections, we can see that numerous H_2_S releasing/stimulating reagents have been developed so far and many of them have been used in cell- or animal-based disease models. However, very few of them have been further advanced into pre-clinic studies, indicating the development of H_2_S as therapeutics is still at the early stage. Nevertheless these studies have revealed interesting sulfur-related biological mechanisms, which can potentially impact future research in this area. In the following section, we intend to review the applications of H_2_S releasing/stimulating reagents in the studies of cancer and glycometabolic disorders, two very common health concerns in the world.

## Hydrogen Sulfide and Cancer

### Effects of H_2_S on Cell Cycle and Proliferation

The cell cycle represents a series of tightly integrated events, which regulate the transition from cellular quiescence to proliferation, and thus ensure high fidelity of the genetic transcript (Schwartz and Shah, 2005). In eukaryotic cells, the cell cycle includes four conventional phases, i.e., Gap phase 1 (G1); DNA synthesis phase (S); Gap phase 2 (G2), during which the cell prepares itself for division, and mitosis phase (M), during which the chromosomes separate and the cell divides. Dysregulation of the cell cycle is an important cause of cellular overproliferation and cancer (Schwartz and Shah, 2005; Malumbres and Barbacid, 2009).

The treatment of oral squamous cell carcinoma cells, Cal27, GNM, and WSU-HN6, with a H_2_S donor, NaHS, significantly downregulated cell cycle regulatory genes, RPA70 and RB1, and upregulated proliferating cell nuclear antigen and cyclin-dependent kinase 4 (CDK4), resulting in cell proliferation (Ma et al., 2015). In human colon cancer HCT 116 cells and hepatocellular carcinoma cells, H_2_S-mediated proliferation was investigated through addition of NaHS and induction of CSE expression, respectively. It was found that the effects of H_2_S on tumorigenesis were associated with the decreased proportion of cells in G0–G1 phase, downregulation of cyclin-dependent kinase inhibitor p21, and with the increase in proportion of S phase cells (Cai et al., 2010; Yin et al., 2012). A recent study showed that by inhibiting endogenous CBS/H_2_S pathway quinolone-indolone conjugate (QIC)2 could significantly attenuate hepatoma cell proliferation (Jia et al., 2017). These findings suggest that H_2_S participates in the development of cancer through shortening cell cycle and inducing proliferation.

On the other hand, some researchers used SPRC as a H_2_S stimulating reagent, and found that the treatment with SPRC could induce cell cycle arrest at G1/S phase and consequently inhibit proliferation of SGC-7901 gastric cancer cells (Ma et al., 2011). The synthetic slow-releasing H_2_S donor, GYY4137, also showed similar anti-proliferation in hepatocellular carcinoma model (Lu et al., 2014). In addition, the exposure to GYY4137 for 8 days can induce cell cycle arrest at the G2/M phase in human breast adenocarcinoma MCF-7 cells (Lee et al., 2011). Some studies introduced H_2_S-releasing moieties to NSAIDs and found that these hybrids have enhanced anti-tumor activity and gastrointestinal (GI) safety compared with the prodrugs (Kodela et al., 2015a,b). Of note, a study indicated that H_2_S-releasing NSAIDs inhibited cellular proliferation in a tissue type-independent manner (Chattopadhyay et al., 2012). TTM also exerts anti-cancer effects through chelating and reducing copper, which can promote angiogenesis and feed an expanding tumor (Beauchamp et al., 1984; Garber, 2015). However, it is still unclear if H_2_S is involved in TTM’s anti-cancer effects.

From these studies, it has been indicated that H_2_S has both pro-proliferation and anti-proliferation, even in the same tumor cells, which seems to be conflicting. Notably, in the reports of H_2_S’s pro-proliferation, the inorganic sulfide salts, such as NaHS and Na_2_S, were usually used. However, in the reports of H_2_S’s anti-proliferation, the slow-releasing H_2_S donors such as GYY4137 and dithiolethione were used. Besides the different H_2_S release profiles, the former donors (e.g., NaHS and Na_2_S) are simple donors, whose byproduct can almost be neglected. However, the latter synthetic donors are structurally complicated and may produce biologically active byproducts after H_2_S release, or the donor molecules themselves can have H_2_S independent activities. Further studies are needed to clarify these effects.

### Effects of H_2_S on Cellular Apoptosis

It is necessary for multicellular organisms to keep a balance between proliferation and apoptotic cell death. If this well controlled balance is disrupted, the development of cancer may be triggered (Cotter, 2009). Induction of apoptosis has become an effective strategy for cancer prevention and treatment (Li et al., 2011; Fernald and Kurokawa, 2013; Fulda, 2015). However, if the apoptosis is suppressed in tumor cells, these cells will survive and even resist chemotherapy. Studies have shown that the treatment with various forms of H_2_S donors could induce cell apoptosis in cancer. For example, the exposure of oral cancer cells to H_2_S gas for 72 h markedly led to apoptotic cell death, but did not affect healthy oral keratinocytes (Murata et al., 2014). It was found that the expression of CSE in human melanoma cells was elevated and the overexpressed CSE provoked spontaneous apoptosis. Importantly, exogenously applied H_2_S donor, DATS, or CSE substrate, L-cysteine, can both inhibit tumor growth in mice (Panza et al., 2015). In addition, the slow-releasing donor GYY4137 could trigger apoptosis of many types of cancer cells, including HeLa, HCT-116, Hep G2, HL-60, MCF-7, MV4-11, and U2OS, causing significant inhibition of tumor growth (Lee et al., 2011).

On the other hand, several reports showed that H_2_S of GI tract could inhibit the phytochemical agent β-phenethyl isothiocyanate-induced cellular apoptosis in human colon cancer HCT116 cells (Rose et al., 2005). Moreover, the application of exogenous H_2_S, in the form of NaHS at concentration of 500 μM, dramatically increased cell viability but decreased the number of apoptotic cells through activation of NF-κB pathway in PLC/PRF/5 hepatoma cells (Zhen et al., 2015).

From these studies, it can be noticed that the effects of H_2_S on cellular apoptosis are also contradictory, similar to its effects on proliferation. Therefore, more related and designed research has become imperative.

### Effects of H_2_S on Angiogenesis

In cancer progression, including tumor growth, invasion, and metastasis, angiogenesis plays a vital role through supplement of oxygen and nutrients (Zhang G. et al., 2013). The proliferation and migration of endothelial cells and fibroblasts are the base for angiogenesis, and this progress is regulated by a number of growth factors, such as VEGF, fibroblast growth factor, and epidermal growth factor (Carmeliet and Jain, 2011; Weis and Cheresh, 2011). H_2_S, in the form of NaHS, could induce VEGF expression and promote angiogenesis in soluble fms-like tyrosine kinase 1 (sFlt1)-mediated renal injury (Holwerda et al., 2014) or in hind limb ischemic rats (Wang et al., 2010). Since VEGF-mediated angiogenesis contributes to cancer progression, H_2_S’s pro-angiogenic activity may cause cancer, which is supported by some recent studies. In colon cancer, H_2_S produced by CBS in tumor can stimulate angiogenesis, increase tumor blood flow and ATP generation, resulting in tumor growth. The pharmacological inhibition of H_2_S generation with a selective CSE inhibitor, aminooxyacetic acid, could impede endothelial cell migration and tumor cell proliferation, migration, and invasion (Szabo and Hellmich, 2013; Szabo et al., 2013). In ovarian cancer, similar H_2_S pro-angiogenic effects on tumor growth were also demonstrated (Bhattacharyya et al., 2013; Hellmich et al., 2015). Besides VEGF induction, H_2_S can activate its receptor VEGFR2 via breaking Cys1045–Cys1024 disulfide bond (Tao et al., 2013). Despite the aforementioned anti-proliferation and pro-apoptosis, H_2_S may be used in cancer therapy. However, its proangiogenesis will impede the use in this field. In combination of VEGF neutralizing antibody or VEGFR2 antagonist may overcome this effect of H_2_S on angiogenesis and contribute to its anti-cancer effects.

## Hydrogen Sulfide and Glycometabolism Disorder

Diabetes mellitus (DM) is another common issue affecting public health. As reported by WHO, the number of patients suffering DM has exceeded 420 million throughout the world. It is noteworthy that diabetic patients can carry carcinoma more easily than healthy people (Ben et al., 2011; Jiang et al., 2011; Xu et al., 2013; Tsilidis et al., 2015). Disorders of glucose metabolism are one of the most important features of DM. A growing body of evidence has shown H_2_S plays an important role in the disordered glucose metabolism (Wallace and Wang, 2015; Untereiner and Wu, 2017). In this section, the effects of H_2_S on glucose metabolism are discussed.

### Effects of H_2_S on Insulin Secretion

In the human body, there are many hormones which can increase blood glucose, such as growth hormone, glucocorticoid, and glucagon. However, insulin is the only hormone that decreases blood glucose. Consequently, insulin’s abnormal secretion or/and dysfunction is the significant reason for glycometabolism disorder. In general, H_2_S can affect insulin-secreting beta cells in two ways, i.e., inhibiting secretion of insulin from the cells (Yang et al., 2005; Tang et al., 2013), and preventing them against various stimuli-induced cellular apoptosis (Lipson et al., 2006; Okamoto et al., 2013).

**FIGURE 11:**
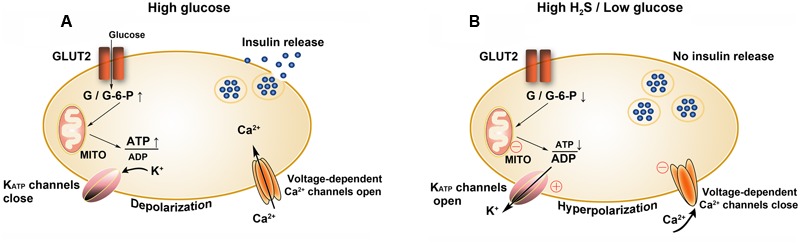
Effects of H_2_S on insulin secretion by beta cells. **(A)** Insulin secretion is stimulated at high glucose levels and **(B)** inhibited at high H_2_S or low glucose levels. GLUT2, glucose transporter type 2; G, glucose; G-6-P, glucose 6-phosphate; MITO, mitochondria; ○, targets of H_2_S; ⊕, to activate; Θ, to inhibit.

It is documented that in streptozotocin (STZ)-induced diabetic rats, the expression of H_2_S synthetases, including CSE and CBS, in the liver and pancreas is significantly upregulated comparing with the control rats. The induction of the two enzymes is restored by the exogenous supplement of insulin (Yusuf et al., 2005). In an *in vitro* study, the administration of H_2_S to INS-1E cells, a beta cell line, obviously attenuates insulin secretion triggered by a high concentration of glucose (Yang et al., 2005). In another beta cell line, HIT-T15 cells, similar findings have been demonstrated. In addition, it is reported that the inhibitory effects of H_2_S on insulin secretion depend on opening K_ATP_ channels (Ali et al., 2007). These studies indicate that under diabetic conditions, the elevated levels of endogenous H_2_S can open K_ATP_ channels in islet beta cell membrane, resulting in hyperpolarization of membrane potential and reduction of insulin secretion (Yang et al., 2005). Besides working as a K_ATP_ channel opener, H_2_S provided by NaHS can directly inhibit L-type voltage-dependent Ca^2+^ channels, thereby reducing insulin secretion in a K_ATP_ channel-independent manner (Tang et al., 2013). In MIN6 cells, a pancreatic beta cell line, through decreasing the intracellular ATP content, the enhancement of H_2_S generation by L-cysteine can also indirectly open K_ATP_ channels and then inhibit insulin secretion (Kaneko et al., 2006). All of these studies manifest that H_2_S inhibits insulin secretion by targeting various links, including inhibition of ATP synthesis, activation of K_ATP_ channels and inactivation of L-type voltage-dependent Ca^2+^ channels, respectively (Wallace and Wang, 2015; **Figure 11**).

Similar to STZ-induced diabetic model, Zucker DM rats have higher endogenous H_2_S content than obese rats. Notably, the inhibition of endogenous H_2_S synthesis in Zucker DM rats improves insulin release and relieves hyperglycemia (Wu et al., 2009). These studies demonstrate that H_2_S can inhibit insulin secretion, and the downregulation of H_2_S system seemingly benefits diabetic prevention and treatment.

Actually, endogenous H_2_S does not always work as a foe. For example, some researchers have shown that H_2_S can prevent islet beta cells from various stimuli-induced injury (Kaneko et al., 2009; Taniguchi and Niki, 2011). CBS is widely expressed in the whole pancreas, while CSE is mainly expressed in the islets of pancreas. In the isolated pancreatic beta cells, basal CSE expression is very low, but can be elevated by the treatment with high concentrations of glucose. Additionally, both exogenous administration of H_2_S (NaHS) and stimulating endogenous H_2_S generation with L-cysteine can impede oxidation-induced cellular apoptosis (Kaneko et al., 2009). Besides excessive apoptotic death of beta cells, the persistently elevated inflammatory response can damage islet cells and cause many diabetic complications. Therefore, DM has been accepted as an inflammatory response disease and thus anti-inflammation is a very important way to treat DM (Donath, 2014, 2016; Esser et al., 2015). Interestingly, the application of NaHS to release H_2_S can significantly inhibit proinflammatory factors-induced injury in primary cultured pancreatic beta cells and MIN6 cells (Taniguchi et al., 2011). In HFD-induced type 2 DM mice, the knockout of CSE (to reduce H_2_S) aggravates oxidation-related insults without promotion of insulin secretion or reduction of blood glucose levels (Okamoto et al., 2013). Our studies indicate that in diabetic skin complications, H_2_S provided by NAC or NSHD-1, a synthetic slow-releasing H_2_S donor, can exert protective effects against diabetes-like injury (Yang et al., 2014, 2017b). However, some other studies have shown that H_2_S participates in ER stress-mediated cellular apoptosis through activation of p38 MAPK pathway (Yang et al., 2007), which is not completely consistent with the report by Taniguchi et al. (2011).

Based on the above studies, it can be summarized that the effects of H_2_S on insulin secretion vary and may be dependent on different stages of diabetic development. At the early stage, hyperglycemia-induced CSE upregulation may be a protective mechanism in the body. The increased H_2_S level can exert anti-oxidation, anti-inflammation, and inhibition of autoimmune response, consequently protecting pancreatic beta cells. With the diabetic development, the content of H_2_S further increases, and the elevated H_2_S will inhibit insulin secretion and then reduce the overload of diabetic beta cells via reduction of ATP content, activation of K_ATP_ channels, or inhibition of L-type voltage-dependent calcium channels (Okamoto et al., 2015). However, if the hyperglycemia state persists, the endogenous content of H_2_S will exceed its threshold, which triggers ER stress response and resultant apoptosis (Yang et al., 2007). Therefore, the regulation of endogenous H_2_S generation is very important, i.e., at the early stage of DM, the administration of H_2_S may be beneficial, while at its late stage inhibiting H_2_S generation may be a reasonable treatment strategy.

### Effects of H_2_S on Hepatic Glucose Metabolism

The liver is an important organ to control blood glucose homeostasis, by balancing glycogen synthesis, to reduce blood glucose levels, and gluconeogenesis, to raise blood glucose levels. Hepatic malfunction can also lead to abnormal glucose metabolism, even diabetic development. Importantly, H_2_S plays a potential role in the glucose metabolism in the liver. In HepG2 hepatocytes, H_2_S (NaHS) can inhibit glucose uptake and glycogen storage through reduction of glucokinase activity (Zhang L. et al., 2013). Additionally, NaHS can induce gluconeogenesis and glycogen breakdown via phosphoenolpyruvate carboxykinase in liver (Untereiner et al., 2016). By these means, H_2_S can increase blood glucose levels. However, it was also found that under physiological conditions, the effects on CSE/H_2_S signal was inhibited by insulin, and perhaps H_2_S does not significantly regulate hepatic glucose output (Zhang L. et al., 2013). When hyperglycemia persists or insulin resistance develops, the CSE/H_2_S signal is obviously activated. A further study showed that the elevated H_2_S content can cause pyruvate carboxylase *S*-sulfhydration and activation, increasing gluconeogenesis and blood glucose levels. Interestingly, the expression of CSE can be increased by glucose deprivation, but decreased by a HFD, indicating that hepatic H_2_S generation is significant to maintain glycemic homeostasis (Ju et al., 2015).

### Effects of H_2_S on Glucose Metabolism of Adipose Tissues

When blood glucose increases, the adipose tissues can store the excessive glucose as body fat. Conversely, when blood glucose decreases, some fat stored in adipose tissues can be mobilized and used for gluconeogenesis and energy. Therefore, the adipose tissues usually serve as another buffer for blood glucose in the body alongside the liver. It was found that CSE is expressed in adipose cells and preadipocytes. Stimulating endogenous H_2_S production with L-cysteine can inhibit glucose uptake by adipose tissues via reducing insulin sensitivity (Feng et al., 2009). A 2-deoxy-[(3)H] glucose uptake experiment *in vivo* showed that the basal and insulin-stimulated glucose uptake by adipose tissues of Kir 6.2(-/-) mice, deficient in the function of K_ATP_ channel, is enhanced compared with that of wild-type mice (Miki et al., 2002), which is supported by an earlier study (Tayo, 1976). As a K_ATP_ agonist, it is reasonable that H_2_S has an opposite effect comparing with the knockout of Kir 6.2. However, Feng et al.’s (2009) study showed that the effects of H_2_S do not depend on K_ATP_ channels, but are rather associated with PI3K signaling pathway. Besides inhibition of glucose uptake, H_2_S is involved in TNFα-induced insulin resistance in 3T3-L1 adipocytes (Huang et al., 2013). Therefore, for the regulation of glucose uptake by adipose tissues H_2_S does not seem to play a beneficial role.

It is worthwhile to note that some recent studies showed the opposite action of H_2_S on glucose regulation by adipose tissues. Manna and Jain (2011, 2012) found that the administration of H_2_S (Na_2_S) or its stimulating agent (L-cysteine), even VD, can enhance the ability of adipose tissues to take up glucose. This effect is associated with increases in insulin sensitivity and GLUT4 activity. In another *in vivo* study, H_2_S (NaHS) was found to promote adipocytes to uptake glucose and thus reduce fasting blood glucose levels and increase glucose tolerance (Xue et al., 2013). Besides the improvement of adipocyte function, H_2_S can protect the adipocytes against high concentration of glucose-induced adipocyte dysfunction, evidenced by the restored monocyte chemotactic protein (MCP)-1 and adiponectin secretion (Pan et al., 2014). In addition, H_2_S can modulate adipogenesis and adipocyte maturation (Tsai et al., 2015). Hence, the roles of H_2_S in glucose homeostasis regulated by adipose tissues are very complicated and even contradictory. The reason for this may be related to the amount of H_2_S, processing time, experimental model, and diabetic development stage.

## Future Directions

### To Develop More Selective CSE/CBS Modulator

In recent years, H_2_S has received much increased attentions in biomedical fields because of its functions in physiological and pathophysiological processes. As discussed above, endogenous H_2_S not only plays many beneficial roles, but also exerts a great number of adverse effects in the body. Thereby, its fine modulation may have great therapeutic significance for some diseases. Genetic approaches are very useful for basal studies in animals or cells, by modulating the deficiency or overexpression of CSE/CBS. However, in clinical trials, pharmacological approaches should be more promising by modulating CSE/CBS expression or activity with a selective agonist/antagonist (Wallace et al., 2009), which is still quite scarce although there are a handful of non-selective ones. Searching for more selective inhibitors or stimulators of CSE/CBS should be attractive for researchers.

### To Develop H_2_S Donor Materials with Targeted Delivery

Another pharmacological approach is what we have mainly focused on in the present article, i.e., H_2_S releasing agents. Studies in this field have made fantastic progress, especially in recent years. Through this article, we can see that present donors have gradually been improved in water-solubility, stability, releasing controllability, and byproduct toxicity. Nevertheless, one should bear in mind that targeted delivery is critically affecting clinical usage. For example, some compounds have rather unique effects in cellular experiments or local administration in animals, while no effects were found when used systemically. Additionally, it is local rather than systemic H_2_S changes that lead to the pathology or disease. Consequently, a rise in local H_2_S content will be more valuable in *ex vivo* application of H_2_S. In this regard, ROS-activated (Zhao and Pluth, 2016) and pH-controlled H_2_S donors (Kang et al., 2016) may partially possess this feature. Targeted delivery of H_2_S donors or materials should be another exciting research topic.

## Conclusion

H_2_S has been considered as one of the endogenous gaseous signaling molecules with various effects in the body. In some pathological process, e.g., cancer and glycometabolic malfunction, different studies show that H_2_S may exert different or even opposite actions. The reasons may be complex or even perplexing; thereby more related studies are still required to reveal the mystery. Nevertheless, reduced endogenous H_2_S has been linked to the cause of many diseases or their stage-dependent injury. Therefore, to enhance endogenous H_2_S content is of great pharmacological significance with the use of H_2_S releasing/stimulating reagents. It is gratifying to see the development of H_2_S releasing reagents has made great progress in recent years. It is anticipated that this will continue to be an active research topic. We expect to see more interesting work arising in this field.

## Author Contributions

All authors listed have made substantial contribution to the work and approved it for publication.

## Conflict of Interest Statement

The authors declare that the research was conducted in the absence of any commercial or financial relationships that could be construed as a potential conflict of interest.
